# Improving knowledge, attitude and practice on norovirus infection diarrhea among staff of kindergartens and schools: a before-after study

**DOI:** 10.1186/s12889-024-19235-w

**Published:** 2024-07-02

**Authors:** Hongxin Lyu, Dongmei Liang, Riyan Luo, Yunlong Feng, Lei Liu, Sixia Yang, Fuling Cai, Zhen Zhang, Huawei Xiong

**Affiliations:** 1 Shenzhen Longhua District Center for Disease Control and Prevention, Shenzhen, China; 2 Bao’an District Public Health Service Center, Shenzhen, China; 3 Fubao Public Health Center, Futian District, Shenzhen, China; 4 Shenzhen Longgang District Nanwan Public Health Service Center, Shenzhen, China; 5 Shenzhen Center for Disease Control and Prevention, Shenzhen, China

**Keywords:** Before-after study, Teacher, Norovirus diarrhea, Knowledge, Attitudes, Practices, Educational intervention

## Abstract

**Background:**

Norovirus gastroenteritis outbreaks were common in schools and kindergartens and were more related to faculty knowledge, attitude, and practice level. Gastroenteritis outbreaks caused by norovirus in educational institutions were the prominent cause of Public Health Emergency Events in China. This study aimed to explore the transformation in the contribution of KAP items related to outbreak prevention before and after intervention and the impact of demography factors on the intervention.

**Methods:**

This study sampled 1095 kindergarten and 1028 school staff in Shenzhen, China. We created a questionnaire consisting of 35 items in 4 parts, and each item was rated on a scale of 1–5 according to the accuracy. Univariate analysis of non-parametric tests and binary logistic regression were used to estimate the score difference on demographic characteristics, each item and KAP. The odds ratios (OR) with 95% confidence and intervals (CI) for the association between statistical indicators were mainly used to explain the effects before and after intervention.

**Results:**

Overall, 98.72% and 74.9% of the kindergarten and school participants were female, and all respondents had the highest scores difference of practice. Following intervention, univariate analysis indicated that primary school and female respondents achieved higher knowledge scores. Staff age beyond 35 (OR = 0.56, CI:0.34–0.92; OR = 0.67, CI:0.50–0.90) and with more than ten years of service (OR = 0.58, CI:0.36–0.91; OR = 0.38, CI:0.17–0.84) demonstrated a significantly lower post-intervention score for attitude and practice in both kindergartens and schools. The staff members exhibited a general lack of familiarity with the transmission of aerosols and the seasonal patterns of NoVs diarrhea pandemics. Item analysis revealed that kindergarten staff aged 26 and above demonstrated superior performance in terms of the efficacy of medical alcohol for inactivation (OR = 1.93, CI:1.13–3.31) and management strategies for unexplained vomiting among students (OR = 1.97, CI:1.21–3.18). Private school personnel displayed more significant improvement in their practices following educational interventions. School administrators' negative attitudes were primarily evident in their perspectives on morning inspections (OR = 0.11, CI:0.05–0.84).

**Conclusions:**

The potential negative impact of faculty age on NoVs-related knowledge can be mitigated by the positive attitudes fostered through seniority. Furthermore, it is imperative to urgently address the lack of knowledge among administrators, and the identification and treatment of vomiting symptoms should be emphasized as crucial aspects of school prevention strategies. Therefore, education authorities should implement comprehensive public health interventions in the future.

**Supplementary Information:**

The online version contains supplementary material available at 10.1186/s12889-024-19235-w.

## Background

Acute gastroenteritis (AGE), being a significant global health concern, exerts a profound impact on the health status of young children in developing nations. Norovirus (NoVs) infection is responsible for causing the second highest burden among all gastroenteritis worldwide [1]. Generally, there has been a gradual increase in the proportion of NoVs-induced gastroenteritis cases among children, with no lasting immunity to NoVs [1]. A study encompassing 45 countries globally revealed that 17.7% of pediatric gastroenteritis cases were attributed to NoVs, thereby urgently highlighting its role as the second leading cause of AGE [2]. Genogroups GI and GII of NoVs primarily contribute to acute gastroenteritis episodes [3], particularly in southern China. Furthermore, the virus's highly contagious nature and survival ability are accountable for its widespread transmission.

Person-to-person transmission is the primary mode of NoVs infection for children aged over two years [4]. Surveys have shown that kindergartens and schools in China are more susceptible to causing Public Health Emergency Events (PHEE) related to Norovirus gastroenteritis [4–6]. Despite the presence of sufficient health education in schools nowadays, with a focus on nutrition, myopia, and other aspects [7], limited propagation has been recorded worldwide on the effectiveness of health education for infectious diseases. Furthermore, there is still room for improvement in terms of educational institutions' compliance with the Norovirus Outbreak Management and Disease Prevention Guidelines (2015 version) issued by China CDC.

In China, the incidence of outbreaks in children is closely associated with the educational background of their caregivers [8]. Research has confirmed that caregivers lacking awareness about the origin, causes, and symptoms of diarrheal diseases are more likely to expose children to pathogens [9]. Teachers and administrators often serve as the primary point of contact during infectious diarrhea epidemics. A study by Li et al. revealed that teachers were unaware of NoVs infection just like parents [10]. Moreover, even educators with better knowledge were insufficient in rectifying improper practices [11]. Teachers who failed to acknowledge the gap between perceived teaching importance and actual teaching performance were at risk of incompetence in their instructional practice [12]. This issue is particularly prominent during the implementation process of health education. School faculty should consult reliable sources such as healthcare professionals or official health organizations for accurate information on NoVs AGE prevention.

The Knowledge, Attitudes, and Practices (KAP) investigation has been widely acknowledged as a reliable approach for assessing health promotion levels [12]. However, existing studies primarily focus on students and parents' KAP rather than faculty members. Community health intervention activities within educational institutions play an indispensable role in prioritizing health issues, devising effective strategies, and implementing them to achieve improved public health outcomes [13]. Furthermore, health promotion initiatives not only enhance the commitment of school administrators in preventing NoVs gastroenteritis at its source but also alleviate the significant societal burden associated with outbreaks [14]. Simultaneously, implementing appropriate health education measures targeting faculty members regarding NoVs gastroenteritis can enhance emergency preparedness and prevent the recurrence of similar incidents [15].

During the implementation of the Health School Plan, we can conduct pre- and post-intervention analyses to identify gaps in knowledge, attitudes, and practices that require attention. By addressing these gaps, we aim to alleviate the challenges faced in managing gastroenteritis outbreaks in schools and significantly reduce norovirus infections among children in the future. Ultimately, this intervention will equip staff with practical tools to enhance their awareness of preventing NoVs-related cluster outbreaks.

## Materials and methods

### Study design and participants

**Fig. 1:**
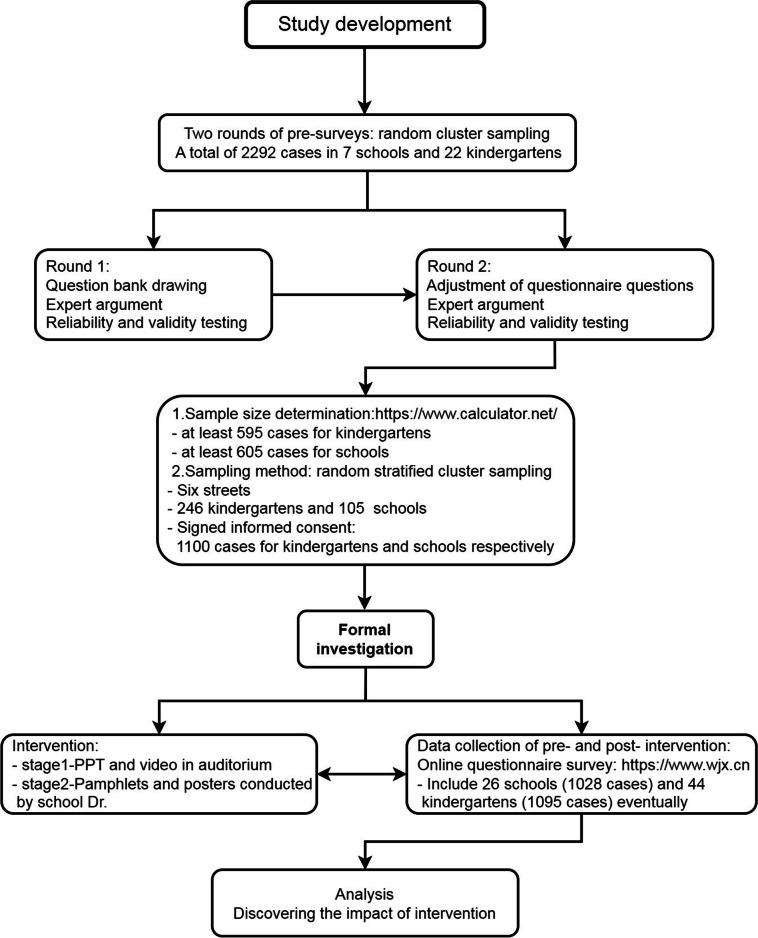
Flow diagram of the study

A prospective before-and-after exploratory study was conducted to evaluate the knowledge, attitudes, and practices (KAP) after an educational intervention for faculty members in kindergartens, primary schools, and secondary schools. The confidence level and allowable error were set at 99% and 5%, respectively, following the single population proportion method [16]. Based on a preliminary survey, the population proportion (including teachers and educational administrators) was estimated to be 75%. Considering an expected dropout rate of 20%, we determined the sample size accordingly. For the formal investigation, a random stratified cluster sampling method was employed. Informed consent forms were signed by a total of 2,200 teachers and staff members from schools and kindergartens (Fig. 1). The participants included both front-line teachers and administrators responsible for administrative management without direct teaching responsibilities. Age groups and length of service were categorized based on local health promotion levels as well as previous studies [17, 18].

### Development of NoVs diarrhea KAP scale and intervention

The survey framework encompasses various components, including knowledge pertaining to NoVs, post-infection epidemiology, case management, opinions on prevention and control policies, as well as epidemic control measures. It consists of 35 items divided into four sections: basic information (7 items), knowledge (8 items), attitude (8 items), and practice (12 items). The structured questionnaire was designed using a Likert scale with five response options: strongly disagree, disagree, neutral, agree, and strongly agree. Scores ranging from 1 to 5 were assigned based on the accuracy of responses.

To enhance the scale's reliability and validity, we conducted two rounds of preliminary surveys. Following adjustments, the survey questionnaire results obtained from schools and kindergartens revealed Cronbach's α values of 0.87 and 0.78, respectively. The Bartlett's sphericity test yielded statistically significant results (*p* < 0.001). Two-stage intervention measures were implemented: firstly, CDC personnel delivered multimedia presentations utilizing PowerPoint and educational videos in each institution's auditorium, with participants required to register upon entry; secondly, after one month of auditorium presentations, the school doctor distributed informational pamphlets and posters regarding NoVs infection to each participant.

### Data collection procedure

In response to the impact of the COVID-19 pandemic, electronic online questionnaires were utilized for data collection before and after intervention. Each respondent was required to complete all questions within a designated timeframe of 5–10 min without utilizing external resources for assistance. All questions were mandatory, necessitating participants to answer them prior to submitting the questionnaire. The data collection team diligently verified the inclusion of cases in order to ensure authenticity and logicality throughout questionnaire completion. Non-compliant questionnaires will be requested for re-completion at each stage of investigation.

### Statistical analysis

We employed non-parametric tests, such as the Mann–Whitney U test and the Kruskal–Wallis test, to examine the disparities in scores following the intervention concerning demographic factors. Utilizing clustering outcomes derived from gray clustering and assessment scores obtained through AHP, we assessed the "good" or "bad" performance of KAP using an 80% threshold [10] of overall scores via ROC curve analysis. Multivariate analysis was conducted using binary logistic regression analysis. The dependent variable was identified as the overall score based on performance, while each question's score was categorized into two groups according to a 4-point cutoff identified as an independent variable. Additionally, each question was treated as a dichotomous dependent variable based on whether the difference between post- and pre-intervention scores exceeded or equaled zero, with school and demographic factors serving as independent variables. The analysis aimed to determine if demographic factors can positively influence specific aspects of KAP content. Regression models were established using backward Wald's test for analyzing variables. Statistical significance was considered at a two-sided *p*-value < 0.05 level. All analyses were performed using IBM SPSS Version 25.0 and R software (Version 4.2.1).

## Results

From 10th January to 7th March 2023, a total of 2123 respondents were included in the analysis, considering an attrition rate of 6.5% and 0.45% for kindergartens and schools respectively due to irregular or random completion (Fig. 1). The majority of respondents were female (kindergarten and school, 98.72% vs. 74.90%). Approximately half of the participants had less than five years of service, with percentages being 46.30% in kindergartens and 40.47% in schools (Table 1).

**Table 1:** Distribution and Score difference of factors of demographic

Variable	Kindergartens (*n* = 1095)		Schools (*n* = 1028)	
	N(%)	Score difference Mean(SD)	N(%)	Score difference Mean(SD)
**Nature of school**^a^				
Public	766(69.95)	9.99(11.15)	425(41.34)	4.07(7.12)
Private	329(30.05)	9.58(10.46)	603(58.66)	5.06(8.17)
**Sex**^a^				
Male	14(1.28)	9.93(11.64)	258(25.10)	3.84(7.71)*
Female	1081(98.72)	9.88(10.94)	770(74.90)	4.92(7.77)*
**Age**^b^				
≤ 25	274(25.02)	9.44(11.13)	133(12.94)	4.28(7.03)
26–35	418(38.17)	9.79(10.41)	539(52.43)	4.56(7.91)
> 35	403(36.81)	10.24(11.35)	356(34.63)	4.93(7.81)
**Position**^b^				
Teachers	668(61.01)	8.57(10.89)	1000(97.28)	4.70(7.81)
Administrators	76(6.94)	10.18(11.78)	28(2.72)	3.04(5.59)
Childminders	351(32.05)	10.18(10.85)	-	-
**Length of service(years)**^b^				
≤ 5	507(46.30)	9.48(10.20)	416(40.47)	4.52(7.63)
6–10	403(36.81)	10.63(11.55)	284(27.63)	4.50(7.77)
> 10	185(16.89)	9.27(11.49)	328(31.90)	4.95(7.93)
**Learning stage**^a^				
Primary schools	-	-	647(62.94)	5.72(8.40)*
Secondary schools	-	-	381(37.06)	2.84(6.13)*

^a^Mann-Whitney U test

^b^Kruskal-Wallis test

^*^p < 0.05

Generally, respondents from kindergartens exhibited the lowest difference in attitude scores (1.2 ± 4.02) and the highest difference in practice scores (2.01 ± 5.48), while schools had the lowest difference in knowledge scores (2.37 ± 6.57) and the highest difference in practice scores (3.83 ± 5.89) (Additional file Table S1). After intervention, primary school respondents scored significantly higher than secondary school respondents in both knowledge (*p* < 0.001) and practice score (*p* = 0.013). Female respondents from schools demonstrated higher knowledge scores compared to males (*p* = 0.018). School teachers displayed a more positive attitude towards NoVs infection prevention compared to administrative personnel (*p* = 0.018). Kindergarten teachers with less than 5 years of service exhibited poorer attitudes towards NoVs infection prevention than those with more than 5 years of service experience (Additional file Table S2).

In the multivariate analysis examining the associations between K, A, P differentials and demographic characteristics of kindergarten faculty, we observed a decreased likelihood of attitude scores (OR = 0.56, 95%CI:0.34–0.92) and practice scores (OR = 0.58, 95%CI:0.36–0.91) among individuals aged over 35 and with more than 10 years of service respectively. Similarly, reduced odds of attitude scores (OR = 0.67, 95%CI:0.50–0.90) and practice scores (OR = 0.38, 95%CI:0.17–0.84) were identified in school staff members within the same age range and years of service (Table 2). However, the analysis revealed a higher likelihood of positive attitude scores when the kindergarten's faculty members have served over 5 years.

**Table 2:** The influence of demographic factors in schools and kindergartens on the effect of KAP intervention

Variables	Kindergarten OR (95%CI)				School OR (95%CI)			
	K	A	P	O	K	A	P	O
**Institution**^1^								
Private	1.04(0.79–1.36)	1.05(0.76–1.44)	0.86(0.63–1.18)	0.88(0.62–1.24)	1.13(0.84–1.53)	1.23(0.92–1.65)	0.98(0.74–1.30)	0.84(0.61–1.15)
**Sex**^2^								
Female	0.65(0.20–2.11)	0.85(0.23–3.13)	1.43(0.44–4.71)	1.38(0.37–5.12)	1.2(0.87–1.67)	0.93(0.67–1.29)	1.09(0.80–1.50)	1.25(0.89–1.76)
**Age**^3^								
26–35	1.32(0.94–1.86)	0.87(0.58–1.31)	0.96(0.64–1.45)	1.36(0.87–2.12)	1.11(0.69–1.78)	1.47(0.94–2.29)	1.14(0.74–1.76)	1.08(0.66–1.76)
> 35	1.26(0.81–1.95)	0.56(0.34–0.92)*	1.10(0.65–1.86)	1.21(0.69–2.14)	1.33(0.72–2.44)	1.23(0.69–2.18)	1.30(0.74–2.27)	1.44(0.77–2.68)
**Post**^4^								
Administrator	1.03(0.61–1.75)	0.99(0.53–1.85)	0.85(0.48–1.51)	0.64(0.34–1.18)	1.7(0.62–4.67)	0.38(0.17–0.84)*	0.75(0.33–1.70)	1.10(0.43–2.85)
Childminder	1.19(0.84–1.69)	1.28(0.85–1.91)	1.20(0.79–1.82)	1.00(0.63–1.59)	-	-	-	-
**Length of service(years)**^5^								
6–10	0.92(0.69–1.23)	1.44(1.03–2.01)*	1.25(0.87–1.79)	0.85(0.58–1.24)	0.84(0.57–1.23)	0.89(0.61–1.30)	1.15(0.80–1.64)	0.90(0.61–1.34)
> 10	0.77(0.51–1.16)	1.83(1.12–2.99)*	0.58(0.36–0.91)*	0.72(0.42–1.22)	0.69(0.43–1.12)	1.19(0.74–1.92)	0.98(0.62–1.54)	0.73(0.44–1.20)
**Learning stage**^6^								
Secondary	-	-	-	-	0.67(0.50–0.90)*	0.90(0.67–1.21)	0.87(0.65–1.15)	0.85(0.62–1.16)

Hosmer–Lemeshow test to identified difference

*p < 0.05. K, A, P and O represent Knowledge, Attitude, Practice and overall items respectively

^1,2,3,4,5,6^means regression references as public institution, male, age ≤ 25, teacher, service years ≤ 5, primary school respectively

### Contribution of questions before and after intervention

Among the kindergarten respondents, the items "NoVs diarrhea outbreaks transmitted by aerosols" (Q2; OR = 1.96, 95%CI:1.17–3.28), "NoVs diarrhea outbreak season" (Q4; OR = 3.71, 95%CI:2.34–5.90), and "The impact of school closure on students" (Q16; OR = 4.72, 95%CI:2.66–8.37) exhibited a significant association with achieving a "good" performance level (*p* < 0.05) prior to intervention implementation. Conversely, the item "Management after sudden vomiting in class" (Q26; OR = 2.51, 95%CI:1.48–4.24) demonstrated a significant contribution (*p* = 0.001) to the overall score improvement in the post-test compared to the pre-test evaluation period (Table 3).

**Table 3:** The influence of each items score on KAP performance before and after intervention

Items	Kindergarten pre-intervention		Kindergarten post-intervention		School pre-intervention		School post-intervention	
	OR (95%CI)	*P*	OR (95%CI)	*P*	OR (95%CI)	*P*	OR (95%CI)	*P*
**Knowledge**								
Q1. Is NoVs most commonly transmitted in schools and kindergartens through the digestive tract?	1.88(1.06–3.32)	0.031	3.31(1.84–5.96)	< 0.001	2.96(1.02–8.55)	0.045	4.52(1.70–12.05)	0.003
Q2. Are NoVs diarrhea outbreaks commonly transmitted by aerosols in schools and kindergartens?	1.96(1.17–3.28)	0.011	-	-	1.70(1.03–2.81)	0.039	-	-
Q3. Can NoVs be transmitted through water?	2.63(1.68–4.14)	< 0.001	3.41(1.83–6.35)	< 0.001	3.15(1.83–5.42)	< 0.001	3.89(1.97–7.71)	< 0.001
Q4. Is summer and autumn have more NoVs related outbreaks in Shenzhen?	3.71(2.34–5.90)	< 0.001	-	-	2.45(1.21–4.95)	0.013	-	-
Q5. Is the average incubation period of NoVs infectious diarrhea 1–2 days?	3.26(1.95–5.44)	< 0.001	2.92(1.47–5.82)	0.002	2.23(1.26–3.95)	0.006	3.06(1.32–7.06)	0.009
Q6. Can medicinal alcohol inactivate NoVs?	2.17(1.39–3.39)	0.001	3.19(1.89–5.40)	< 0.001	1.61(0.99–2.62)	0.055	3.71(2.21–6.22)	< 0.001
Q7. Is vaccination effective in preventing NoVs-infected diarrhea?	2.31(1.49–3.58)	< 0.001	3.73(2.33–5.99)	< 0.001	2.89(1.58–5.28)	0.001	4.11(2.42–6.98)	< 0.001
Q8. Will virus excretion in the short term after the symptoms of diarrhea caused by NoVs have resolved?	2.80(1.64–4.77)	< 0.001	5.69(2.33–13.90)	< 0.001	-	-	2.60(1.22–5.58)	0.014
**Attitude**								
Q9. Are you interested in learning about NoVs infectious diarrhea?	-	-	-	-	-	-	4.22(1.46–12.19)	0.008
Q10. The role of faculty in epidemic prevention and control (non-treatment) is second only to that of school doctors	3.40(2.20–5.26)	< 0.001	2.42(1.48–3.95)	< 0.001	-	-	-	-
Q11. It is necessary for teachers to disclose the epidemic information to parents at any time when the epidemic occurs	3.87(2.23–6.71)	< 0.001	1.81(1.04–3.15)	0.035	-	-	3.63(1.84–7.14)	< 0.001
Q12. The role of morning check in the prevention of infectious diseases is general	2.62(1.65–4.16)	< 0.001	2.61(1.55–4.38)	< 0.001	3.75(2.50–5.61)	< 0.001	4.49(2.68–7.50)	< 0.001
Q13. Faculty members (except school doctors) need to identify the epidemic in time	-	-	-	-	-	-	-	-
Q14. Do you agree that teachers or childminders are the first responders to NoVs diarrhea outbreaks?	3.62(1.89–6.91)	< 0.001	8.06(2.28–28.54)	0.001	2.33(1.51–3.60)	< 0.001	2.10(1.21–3.65)	0.009
Q15. The school closure guideline for epidemic prevention and control are too strict	2.65(1.71–4.09)	< 0.001	3.85(2.41–6.15)	< 0.001	1.82(1.19–2.79)	0.006	4.14(2.47–6.94)	< 0.001
Q16. The impact of school suspension on students is greater than the disease itself	4.72(2.66–8.37)	< 0.001	-	-	2.71(1.73–4.22)	< 0.001	-	-
**Practice**								
Q17. When parents have objections to school suspension, will you take the initiative to explain the guideline?	-	-	-	-	3.09(0.85–11.29)	0.088	-	-
Q18. Will you take the initiative to point out the improperly dispose of vomit and excrement?	-	-	-	-	-	-	18.11(1.71–191.50)	0.016
Q19. When parents state their children have vomiting or diarrhea at home, will you inform them to take their child home for observation?	-	-	-	-	4.90(1.53–15.68)	0.007	-	-
Q20. When there is a cluster epidemic in the class, do you need to take the initiative to discuss with the school doctor whether to suspend classes?	8.11(1.23–53.46)	0.030	8.23(0.92–74.02)	0.060	-	-	-	-
Q21. Is the correct way to deal with the vomit of students in the class to wipe it before disinfecting it?	3.83(2.35–6.25)	< 0.001	3.12(1.72–5.64)	< 0.001	3.12(2.06–4.72)	< 0.001	4.39(2.68–7.19)	< 0.001
Q22. When a diarrhea outbreak occurs, will you communicate with parents about your child's condition?	-	-	-	-	0.17(0.04–0.72)	0.017	-	-
Q23. Will you judge whether the children coming to school meet the quarantine time according to the absence record?	3.19(1.80–5.66)	< 0.001	5.34(1.75–16.29)	0.003	2.60(1.36–4.95)	0.004	-	-
Q24. When a student has unexplained vomiting, do you need to notify parents to take them back?	-	-	-	-	4.05(1.43–11.43)	0.008	9.54(1.11–82.26)	0.040
Q25. Do you pay more attention to students' gastrointestinal symptoms than respiratory symptoms in the morning check?	2.25(1.40–3.62)	0.050	1.71(0.99–2.95)	0.053	-	-	2.21(1.17–4.17)	0.015
Q26. Will you let the student stay in the class after a sudden vomiting?	1.56(0.94–2.59)	0.082	2.51(1.48–4.24)	0.001	2.92(1.93–4.42)	< 0.001	3.03(1.80–5.130)	< 0.001
Q27. Can students infected with NoVs be allowed to return to school 48 h after symptoms disappear?	2.06(1.38–3.08)	< 0.001	1.70(1.11–2.62)	0.015	2.40(1.53–3.76)	< 0.001	3.32(2.02–5.46)	< 0.001
Q28. Will you report the situation to the school doctor when you know that more than one classes have children with diarrhea and vomiting during the same period?	-	-	-	-	-	-	-	-

The '-' indicates the variable exclude from the model. Hosmer–Lemeshow test to identified difference,*p < 0.05. Sub-items with score below 4 were taken as references in independent variables

Among the school's respondents, significant associations (*p* < 0.05) were found between "NoVs diarrhea outbreaks transmitted by aerosols" (Q2; OR = 1.70, 95%CI:1.03–2.81), "NoVs diarrhea outbreak season" (Q4; OR = 2.45, 95%CI:1.21–4.95), "The impact of school closure on students" (Q16; OR = 2.71, 95%CI:1.73–4.22), "School response after learning that children have diarrhea at home" (Q19; OR = 4.90, 95%CI:1.53–15.68), and "Use absence records to determine school closure criteria" (Q23; OR = 2.60, 95%CI:1.36–4.95) and their performance before intervention was rated as "good". Furthermore, the following items showed a significant association with higher scores (*p* < 0.05) after intervention in schools: "Inactivation effect of medical alcohol" (Q6; OR = 3.71, 95%CI:2.21–6.22), "The virus expulsion rule" (Q8; OR = 2.60, 95%CI:1.22–5.58), "Interest in learning about NoVs-diarrhea" (Q9; OR = 4.22, 95%CI:1.46–12.19), "The epidemic information disclosure" (Q11; OR = 3.63, 95%CI:1.84–7.14), "Correction of inappropriate vomit disposal" (Q18; OR = 18.11, 95%CI:1.71–191.50), and "Pay attention to gastrointestinal symptoms during morning check" (Q25; OR = 2.21, 95%CI:1.17–4.17)(Table 3).

### Differences in demographic factors reflected in the interventions

The analysis of demographic influences on kindergarten respondents revealed that private kindergarten respondents exhibited a higher acceptance of interventions compared to public kindergarten respondents in terms of knowledge regarding the typical transmission route (OR = 1.59; *p* = 0.001). However, private kindergarten respondents displayed lower acceptance towards education on the aerosols route of norovirus transmission (OR = 0.61; *p* = 0.002). Faculty members aged 35 and above, as well as those aged between 26 and 35, demonstrated better performance in terms of the inactivation effect of medicinal alcohol (OR = 1.93; *p* = 0.016) and management strategies for disposing unexplained vomiting incidents among students (OR = 1.97; *p* = 0.006), when compared to individuals below the age of 26. When considering teachers as a reference point, childminders exhibited greater awareness regarding virus expulsion timing (OR = 1.55; *p* = 0.045), while administrators were more prone to neglect communication concerning class closure (OR = 0.51; *p* = 0.047) and initiation timing for quarantine measures(OR = 0.46; *p* = 0.025)(Table 4).

**Table 4:** The influence of kindergarten faculty demographic factors on KAP score of each items after intervention

Items	Institution (Public as Ref.)	Sex (Male as Ref.)	Age (≤ 25 as Ref.)		Post (teacher as Ref.)		Length of service (≤ 5 years as Ref.)	
	Private	Female	26–35	> 35	Administrator	Childminder	6–10 years	> 10 years
Q1	1.59(1.22–2.07)**	1.21(0.40–3.69)	1.37(0.98–1.92)	1.26(0.81–1.95)	0.85(0.50–1.45)	0.77(0.55–1.09)	0.79(0.59–1.05)	0.78(0.52–1.19)
Q2	0.61(0.44–0.83)**	1.77(0.54–5.82)	1.02(0.67–1.56)	0.93(0.54–1.60)	0.72(0.38–1.36)	0.87(0.57–1.33)	1.18(0.82–1.68)	1.28(0.73–2.15)
Q3	1.00(0.73–1.36)	0.57(0.13–2.60)	0.99(0.67–1.46)	1.18(0.71–1.96)	1.06(0.57–1.99)	0.91(0.61–1.35)	0.92(0.66–1.29)	0.90(0.56–1.45)
Q4	1.44(1.10–1.87)**	0.53(0.17–1.60)	1.23(0.88–1.72)	1.14(0.74–1.75)	0.98(0.59–1.65)	1.00(0.71–1.40)	0.76(0.57–1.00)	1.04(0.70–1.56)
Q5	0.85(0.62–1.17)	1.05(0.29–3.84)	1.18(0.78–1.76)	0.91(0.54–1.51)	1.21(0.65–2.25)	1.22(0.81–1.83)	1.03(0.73–1.45)	0.77(0.48–1.23)
Q6	0.82(0.60–1.13)	1.54(0.47–5.04)	1.46(0.98–2.18)	1.93(1.13–3.31)*	0.70(0.37–1.33)	0.87(0.57–1.35)	1.14(0.80–1.63)	1.18(0.69–2.00)
Q7	0.60(0.45–0.79)**	0.83(0.26–2.71)	0.92(0.65–1.31)	1.15(0.73–1.83)	0.89(0.51–1.55)	1.38(0.96–2.00)	0.83(0.62–1.13)	1.18(0.76–1.85)
Q8	0.91(0.65–1.26)	1.08(0.29–3.97)	0.61(0.39–0.95)*	0.53(0.31–0.92)*	1.04(0.57–1.93)	1.55(1.01–2.38)*	1.23(0.85–1.77)	0.96(0.59–1.55)
Q9	0.88(0.62–1.24)	2.00(0.61–6.55)	0.82(0.52–1.28)	0.74(0.41–1.32)	1.22(0.63–2.34)	1.41(0.90–2.22)	1.07(0.73–1.56)	0.71(0.43–1.17)
Q10	1.56(1.19–2.04)**	1.43(0.49–4.20)	1.10(0.79–1.54)	0.99(0.64–1.52)	0.70(0.42–1.17)	1.31(0.90–1.85)	1.04(0.79–1.39)	1.00(0.67–1.50)
Q11	0.93(0.69–1.26)	0.46(0.10–2.11)	0.89(0.61–1.32)	0.87(0.53–1.43)	0.68(0.38–1.20)	0.99(0.67–1.46)	1.36(0.98–1.89)	1.43(0.90–2.29)
Q12	0.73(0.54–0.98)*	0.91(0.25–3.33)	0.89(0.61–1.31)	0.91(0.56–1.50)	1.43(0.75–2.73)	1.04(0.70–1.53)	1.05(0.76–1.45)	1.12(0.70–1.79)
Q13	0.90(0.62–1.29)	1.55(0.42–5.70)	0.96(0.60–1.53)	0.85(0.47–1.54)	1.06(0.51–2.20)	0.99(0.62–1.58)	1.56(1.04–2.34)*	1.20(0.69–2.07)
Q14	1.09(0.78–1.53)	0.59(0.13–2.72)	0.71(0.46–1.10)	0.73(0.42–1.26)	0.78(0.42–1.45)	1.02(0.67–1.56)	1.88(1.30–2.73)**	1.24(0.76–2.02)
Q15	0.93(0.69–1.24)	1.70(0.56–5.18)	1.31(0.90–1.89)	0.94(0.58–1.50)	1.41(0.75–2.67)	1.01(0.69–1.48)	0.92(0.67–1.25)	1.25(0.78–1.99)
Q16	0.99(0.71–1.37)	0.98(0.27–3.59)	0.75(0.50–1.13)	0.86(0.51–1.46)	1.22(0.62–2.44)	1.24(0.82–1.90)	1.10(0.78–1.55)	1.67(0.98–2.83)
Q17	0.65(0.46–0.92)*	0.48(0.06–3.75)	1.03(0.65–1.64)	1.03(0.57–1.86)	1.11(0.54–2.30)	1.00(0.63–1.60)	0.87(0.59–1.29)	0.81(0.47–1.41)
Q18	0.75(0.51–1.09)	0.54(0.07–4.19)	1.01(0.63–1.63)	1.48(0.78–2.82)	1.30(0.58–2.95)	1.11(0.67–1.85)	0.88(0.58–1.34)	0.77(0.42–1.39)
Q19	0.83(0.56–1.23)	0.53(0.07–4.14)	1.34(0.82–2.20)	1.74(0.90–3.37)	0.61(0.29–1.30)	0.77(0.45–1.29)	1.28(0.82–2.00)	1.07(0.57–2.00)
Q20	0.90(0.62–1.31)	1.69(0.46–6.26)	0.88(0.54–1.43)	1.37(0.72–2.60)	0.51(0.27–0.99)*	0.71(0.44–1.16)	1.00(0.66–1.51)	0.81(0.46–1.43)
Q21	0.89(0.64–1.24)	1.65(0.51–5.40)	1.06(0.70–1.60)	1.35(0.78–2.33)	0.94(0.49–1.80)	0.98(0.63–1.51)	1.45(1.00–2.09)	0.93(0.57–1.54)
Q22	0.95(0.63–1.41)	0.56(0.07–4.41)	1.17(0.69–1.96)	1.21(0.63–2.34)	0.54(0.27–1.11)	0.71(0.43–1.20)	0.92(0.60–1.43)	0.92(0.50–1.70)
Q23	0.80(0.56–1.13)	0.88(0.19–4.05)	0.93(0.58–1.49)	0.75(0.42–1.34)	0.69(0.36–1.33)	0.93(0.59–1.46)	1.07(0.73–1.58)	1.10(0.64–1.89)
Q24	0.76(0.52–1.09)	1.09(0.24–5.04)	1.97(1.21–3.18)**	1.84(1.00–3.41)	0.46(0.23–0.91)*	0.62(0.38–1.01)	0.88(0.59–1.33)	0.94(0.52–1.72)
Q25	1.28(0.98–1.67)	1.16(0.38–3.52)	1.23(0.88–1.73)	1.38(0.89–2.13)	0.66(0.38–1.15)	0.78(0.55–1.10)	0.77(0.58–1.03)	0.68(0.45–1.03)
Q26	0.69(0.51–0.94)*	1.99(0.65–6.09)	0.96(0.64–1.43)	0.87(0.52–1.45)	1.03(0.56–1.90)	1.15(0.76–1.72)	1.35(0.96–1.90)	1.04(0.65–1.67)
Q27	1.31(0.98–1.76)	0.58(0.16–2.13)	0.95(0.66–1.36)	1.12(0.71–1.79)	1.16(0.66–2.03)	1.02(0.71–1.46)	1.14(0.84–1.55)	0.76(0.50–1.16)
Q28	0.70(0.47–1.05)	0.65(0.08–5.10)	1.28(0.75–2.18)	1.33(0.67–2.63)	0.63(0.29–1.34)	0.77(0.45–1.32)	1.01(0.64–1.59)	0.89(0.47–1.68)

The Odds ratios and 95% confidence intervals for each items. Hosmer–Lemeshow test to identified difference

^*^p < 0.05

^**^p < 0.01

Respondents from private schools exhibited a higher level of receptiveness towards intervention knowledge compared to public schools, including factors such as the average incubation period (OR = 1.60, *p* = 0.002), the inactivation effect of medical alcohol (OR = 1.55, *p* = 0.007), and the limitations of vaccines in prevention (OR = 1.37, *p* = 0.005). Female participants also recognized the crucial role played by faculty members in epidemic prevention (OR = 1.65, *p* = 0.002). Staff aged 26 or above demonstrated lower levels of understanding regarding the transmission of norovirus through water (OR = 0.58, *p* = 0.011; OR = 0.41, *p* = 0.001) and actively explaining suspension guidelines to parents (OR = 0.47, *p* = 0.007; OR = 0.31, *p* = 0.001) compared to those below the age of 26 years old. In contrast to staff with less than five years of service experience, those with more than ten years displayed limited awareness regarding NoVs diarrhea epidemic season (OR = 0.33, *p* = 0.004), the effectiveness of medicinal alcohol (OR = 0.55, *p* = 0.022), and vaccine efficacy(OR = 0.48, *p* = 0.021). Furthermore, the significance of morning checks (OR = 0.42, *p* = 0.03) and gastrointestinal symptoms was often overlooked by school administrators. Conversely, following the intervention, secondary school faculty demonstrated enhanced proficiency in managing vomiting students (OR = 1.77, *p* < 0.001) and implementing NoVs infection quarantine measures for extended durations (OR = 1.67, *p* = 0.002)(Table 5).

**Table 5:** The influence of school faculty demographic factors on KAP score of each items after intervention

Items	Institution (Public as Ref.)	Sex (Male as Ref.)	Age (≤ 25 as Ref.)		Length of service (≤ 5 years as Ref.)		Post (teacher as Ref.)	Type of school (Primary as Ref.)
	Private	Female	26–35	> 35	6–10 years	> 10 years	Administrator	Secondary school
Q1	0.74(0.55–0.99)*	1.37(1.00–1.88)	1.16(0.74–1.81)	1.09(0.62–1.91)	1.00(0.70–1.44)	1.02(0.65–1.61)	2.13(0.78–5.83)	1.05(0.79–1.41)
Q2	1.21(0.89–1.65)	1.03(0.73–1.46)	1.35(0.84–2.16)	1.56(0.84–2.88)	0.93(0.63–1.39)	0.87(0.52–1.44)	1.67(0.55–5.00)	0.71(0.52–0.96)*
Q3	1.37(1.03–1.84)*	0.84(0.61–1.15)	0.58(0.37–0.88)*	0.41(0.23–0.72)**	0.87(0.60–1.26)	1.19(0.75–1.89)	2.05(0.92–4.57)	1.02(0.77–1.37)
Q4	1.16(0.73–1.83)	1.07(0.65–1.75)	1.19(0.5–2.84)	1.12(0.41–3.10)	0.68(0.34–1.36)	0.33(0.15–0.70)**	0.42(0.16–1.09)	0.56(0.35–0.88)*
Q5	1.60(1.18–2.16)**	0.83(0.60–1.16)	0.97(0.62–1.54)	0.55(0.30–1.00)	0.87(0.60–1.27)	1.42(0.88–2.28)	1.42(0.61–3.31)	0.96(0.72–1.30)
Q6	1.55(1.13–2.12)**	0.80(0.55–1.15)	0.69(0.40–1.20)	0.88(0.45–1.76)	0.80(0.52–1.21)	0.55(0.33–0.92)*	0.58(0.25–1.33)	0.98(0.71–1.36)
Q7	1.72(1.17–2.51)**	0.74(0.47–1.16)	0.71(0.35–1.46)	0.73(0.31–1.73)	0.78(0.46–1.33)	0.48(0.26–0.90)*	0.63(0.25–1.60)	0.97(0.65–1.44)
Q8	1.37(1.03–1.83)*	1.07(0.78–1.48)	0.73(0.47–1.12)	0.64(0.37–1.12)	0.96(0.67–1.37)	1.08(0.69–1.71)	0.72(0.28–1.84)	1.09(0.82–1.45)
Q9	1.25(0.90–1.75)	0.61(0.41–0.91)*	0.53(0.30–0.95)*	0.55(0.27–1.14)	0.97(0.65–1.45)	1.30(0.76–2.23)	0.99(0.33–3.01)	0.76(0.54–1.06)
Q10	0.92(0.69–1.28)	1.65(1.21–2.25)**	0.99(0.63–1.55)	0.97(0.55–1.71)	1.06(0.74–1.53)	0.98(0.62–1.54)	2.71(0.99–7.42)	1.37(1.03–1.84)*
Q11	0.91(0.68–1.23)	1.00(0.71–1.41)	1.35(0.86–2.13)	1.29(0.70–2.32)	0.96(0.65–1.40)	1.04(0.64–1.69)	0.84(0.35–1.99)	1.33(0.97–1.80)
Q12	1.09(0.82–1.45)	1.14(0.83–1.56)	1.38(0.89–2.16)	1.86(1.05–3.31)*	0.94(0.65–1.37)	0.64(0.40–1.02)	0.42(0.19–0.92)*	0.69(0.52–0.92)*
Q13	1.28(0.91–1.79)	0.80(0.54–1.18)	1.32(0.80–2.20)	1.25(0.64–2.44)	1.06(0.68–1.64)	1.11(0.64–1.95)	0.82(0.29–2.28)	0.70(0.50–0.99)*
Q14	1.17(0.87–1.56)	0.80(0.57–1.12)	1.17(0.74–1.84)	0.89(0.50–1.58)	0.72(0.50–1.04)	1.23(0.77–1.98)	1.25(0.49–3.22)	1.07(0.80–1.44)
Q15	0.82(0.61–1.10)	1.28(0.93–1.76)	1.15(0.72–1.82)	1.01(0.57–1.80)	0.92(0.63–1.33)	0.84(0.53–1.34)	0.82(0.36–1.87)	0.93(0.70–1.25)
Q16	0.86(0.64–1.17)	1.24(0.89–1.73)	1.36(0.85–2.17)	1.77(0.97–3.22)	0.86(0.58–1.27)	0.71(0.44–1.16)	0.59(0.26–1.34)	0.79(0.58–1.07)
Q17	1.35(0.92–1.98)	0.72(0.48–1.08)	0.47(0.27–0.81)**	0.31(0.15–0.64)**	1.57(0.96–2.57)	2.01(1.10–3.69)*	0.20(0.03–1.52)	1.09(0.75–1.58)
Q18	1.62(1.09–2.41)*	0.62(0.41–0.94)*	0.59(0.34–1.02)	0.43(0.21–0.89)*	0.94(0.58–1.53)	1.11(0.60–2.06)	0.24(0.03–1.83)	0.94(0.64–1.38)
Q19	1.47(1.00–2.15)*	0.79(0.53–1.18)	0.65(0.38–1.11)	0.42(0.21–0.87)*	1.15(0.73–1.82)	1.18(0.65–2.15)	0.27(0.04–2.03)	1.52(1.06–2.18)*
Q20	1.63(1.09–2.44)*	0.72(0.47–1.10)	0.61(0.35–1.05)	0.44(0.21–0.91)*	0.92(0.56–1.50)	1.07(0.57–2.00)	0.59(0.13–2.63)	1.17(0.80–1.72)
Q21	1.69(1.28–2.24)**	0.93(0.67–1.28)	1.09(0.69–1.71)	1.20(0.68–2.13)	0.73(0.50–1.05)	0.65(0.41–1.04)	0.89(0.40–2.00)	1.43(1.07–1.92)*
Q22	1.60(1.03–2.49)*	0.78(0.49–1.23)	0.69(0.38–1.27)	0.56(0.25–1.24)	1.06(0.63–1.77)	0.91(0.46–1.10)	0.36(0.05–2.79)	0.96(0.63–1.47)
Q23	1.43(1.04–1.96)*	0.93(0.65–1.32)	0.63(0.39–1.01)	0.46(0.25–0.85)*	1.06(0.71–1.59)	1.42(0.86–2.35)	0.62(0.21–1.85)	1.15(0.84–1.57)
Q24	1.73(1.17–2.55)**	0.91(0.60–1.37)	0.76(0.43–1.34)	0.65(0.31–1.34)	1.17(0.74–1.87)	1.25(0.69–2.25)	0.80(0.23–2.81)	1.30(0.90–1.87)
Q25	1.31(0.97–1.77)	0.76(0.55–1.05)	0.73(0.47–1.13)	0.47(0.27–0.85)*	1.08(0.75–1.56)	1.14(0.71–1.85)	0.11(0.05–0.84)*	1.00(0.75–1.35)
Q26	1.48(1.13–1.94)**	0.78(0.57–1.08)	0.68(0.43–1.07)	0.72(0.41–1.26)	1.00(0.70–1.43)	0.59(0.38–0.92)*	0.95(0.42–2.11)	1.77(1.33–2.37)**
Q27	1.12(0.83–1.52)	1.23(0.87–1.72)	0.75(0.45–1.26)	0.72(0.38–1.35)	0.91(0.61–1.34)	0.85(0.52–1.39)	0.77(0.34–1.75)	1.67(1.21–2.31)**
Q28	1.74(1.10–2.77)*	0.78(0.48–1.27)	0.66(0.36–1.23)	0.45(0.19–1.03)	0.78(0.44–1.36)	1.03(0.51–2.09)	0.41(0.05–3.14)	1.02(0.66–1.58)

The Odds ratios and 95% confidence intervals for each items. Hosmer–Lemeshow test to identified difference

^*^p < 0.05

^**^p < 0.01

## Discussion

The present study aimed to evaluate the impact of educational institution faculty on the knowledge, attitudes, and practices (KAP) regarding NoVs infectious diarrhea, with a focus on identifying preventive measures for large-scale outbreaks during the resumption of classes following the COVID-19 emergency response. Notably, without considering potential interactions, primary school teachers demonstrated superior educational outcomes compared to other groups, which could be attributed to their higher exposure to NoVs-related outbreaks [19]. In line with previous research [20, 21], female staff exhibited better retention of knowledge than their male counterparts. Furthermore, school teachers displayed a more positive attitude towards health management due to their direct interaction with students; however, it is important to note that experience may have influenced these attitudes [22], potentially explaining the observed variation in scores.

After controlling for all demographic factors in the analysis, the differences in knowledge, attitude, and practice among kindergarten participants were found to be significantly associated with age and length of service. Specifically, individuals over 35 years old exhibited lower levels of attitude towards disease control after intervention, while those who had worked for more than 10 years demonstrated reduced practice. Interestingly, respondents from kindergartens with more than 5 years of service showed a greater inclination towards having faith in disease control following the lecture, possibly due to their confidence in health education abilities [23]. Moreover, teachers displayed a stronger interest in NoVs-diarrhea outbreak prevention compared to administrators; this discrepancy may partly stem from periodic education provided by health authorities. Additionally, post-intervention knowledge scores among staff members at secondary schools were not as favorable as those observed among primary school staff (Table 2).

One of the strengths of this study lies in its specific examination of the benefits derived from Knowledge, Attitude, and Practice (KAP) intervention in preventing NoVs-induced diarrhea. This is evident through the refinement of sensible items. The pre-intervention outcomes indicated that teaching staff had limited familiarity with both the transmission mechanism and epidemic season, a finding consistent with previous studies conducted in Guangdong [24] and Shanghai [25], China. Such lack of knowledge poses a significant challenge for educational institutions to proactively respond to infectious diseases. Furthermore, our study revealed that staff members were initially hesitant to acknowledge the necessity for class suspension prior to intervention, as supported by findings from a Pondicherry study [26].

Attributed to fewer outbreaks in schools compared to kindergartens in southern China [27], school staff have limited understanding of proper case handling and accurate absence record-keeping. Their responses indicate an ambiguous consciousness of precise prevention measures for NoVs related outbreaks [28], including inadequate management of virus exposure environments and patient isolation. However, the intervention significantly boosted the KAP scores of school staff. In terms of knowledge scores, those who agreed that medical alcohol cannot inactivate NoVs and that NoVs can continue to be excreted after recovery scored 3.71 times and 2.6 times higher than those who did not agree after educational sessions. Furthermore, the intervention substantially enhanced the practical scores of school staff, which may account for improvements in information disclosure, appropriate vomit disposal practices, and morning check concerns.

We present demographic differences for each item because existing studies rarely generate targeted recommendations for NoVs diarrhea interventions based on population classification. The results depicted that the variation in intervention effects between public and private kindergartens primarily revolves around knowledge of the disease. Among kindergarten respondents, age has a more significant impact on improvement in emergency experience than length of service. Staff performance is characterized by higher scores in practical content but a need for understanding knowledge of virus excretion as they surpass the age of 26. Additionally, interventions in kindergartens also exhibit notable post characteristics. Childminders appear to be more concerned about virus transmission, while administrators are more inclined to overlook suspension guidelines (Table 4).

In agreement with the findings of kindergartens, the intervention significantly enhanced the knowledge of staff members from private schools. However, a notable distinction was observed as private school staff exhibited a more significant improvement in their understanding and application of practice contents after the intervention, particularly in relation to vomiting treatment, similar to secondary school staff. Despite this progress, it was evident that school staff aged 26 and above still displayed unfamiliarity with preventive disposal measures. Although those with over 10 years of experience were more inclined to explain suspension guidelines (OR = 2.01, 95%CI:1.10–3.69), they encountered difficulties due to age-related learning barriers. Analysis of school administrators revealed that negative attitudes primarily manifested in their perspectives on morning inspections (Table 5). Furthermore, secondary school faculty demonstrated greater emphasis on addressing issues related to sick students rather than NoVs-related knowledge.

Our research has identified some enduring patterns in the prevention and control of NoVs-related diarrhea. Firstly, it is observed that even veteran teachers may impede knowledge dissemination; however, as work experience increases, this limitation can be compensated by a positive attitude towards classroom experiences [29]. Secondly, similar to parents [10], most staff members lack accurate understanding regarding the duration and symptoms of the disease, which may lead to an increased possibility of children attending classes while ill. Finally, Improper handling of children's vomit can undoubtedly lead to secondary transmission [3]. Therefore, it is crucial to pay close attention to students with gastrointestinal symptoms during morning screenings in order to minimize the occurrence of vomiting in classrooms and prevent large-scale outbreaks caused by NoV infections [30]. Moreover, To enhance administrators' attitudes, there is a need for a more compelling program that emphasizes the importance of preventing disease outbreaks on campus through a combination of publicity projects and factual information. Health authorities should prioritize education of administrative managers in both kindergartens and schools to minimize conflicting priorities and provide support for teachers in epidemic prevention efforts [31].

After identifying the corresponding demographic factors influences, it is imperative for us to address the adverse effects of these factors through targeted interventions in future measures. The implementation of comprehensive health interventions at a large scale within educational institutions not only plays a pivotal role in fostering academic achievement but also significantly enhances the well-being of students. We can envision an value-for-money framework [32] that establishes school-based curricula encompassing pertinent measures from the healthcare sector, while concurrently collecting data on the economic burden and returns associated with such initiatives. By incorporating these considerations into public budgets, we can assess the cost-effectiveness of implementing school-based programs. Therefore, this study is of utmost importance in establishing a school-based curriculum and laying the groundwork for enhanced communication with the education department.

This research changed the current limitations of cross-sectional analysis in KAP surveys of infectious diseases. However, it should be noted that the representativeness may be influenced by the unique characteristics of the industry, such as a majority of female participants. The exclusion of doctors from institutions as a prevention and control vulnerability is justified due to their extensive training by the CDC. Additionally, a comparative analysis was not conducted for schools and kindergartens due to differences in their daily awareness-raising and educational measures.

## Conclusion

Based on the post-intervention items, the contribution suggests that the intervention had a significant effect on KAP transformation among faculty members. The impact of interventions was more prominent in improving faculty practices and varied by position, institution, and age. Educational administrators should enhance their ability to assimilate and interpret public health information to improve attitudes towards disease prevention and control. Education authorities should focus on addressing staff weaknesses in KAP to prevent outbreaks of NoVs diarrhea.

### Supplementary Information

Supplementary Material 1.

## Data Availability

The data are available on reasonable request from the first author.

## Abbreviations

KAP: Knowledge, Attitude, and Practice
CDC: Centers for Disease Control
NoVs: Norovirus
AHP: Analytic Hierarchy Process
ROC: Receiver Operating Characteristic Curve
