# Supplementary material for: Improving knowledge, attitude and practice on norovirus infection diarrhea among staff of kindergartens and schools: a before-after study
Source: BMC Public Health. 2024 Jul 2;24:1755. doi: 10.1186/s12889-024-19235-w (PMC11218083; doi:10.1186/s12889-024-19235-w)
Supplement: Supplementary file 1 — Supplementary Material 1. [file 12889_2024_19235_MOESM1_ESM.docx]

| **Table S1** KAP scores of school and kindergarten faculties before and after intervention | | | | | | |
| --- | --- | --- | --- | --- | --- | --- |
| items | Before (S) | **After** (S) | **D-value** (S) | Before (K) | **After** (K) | **D-value** (K) |
| Q1. Is NoV most commonly transmitted in schools and kindergartens through the digestive tract? | 1.74 (0.95) | 1.67 (1.00) | -0.07 (1.26) | 3.45 (1.47) | 2.60 (1.61) | -0.85 (2.64) |
| Q2. Are NoV diarrhea outbreaks commonly transmitted by aerosols in schools and kindergartens? | 3.93 (1.17) | 4.18 (1.10) | 0.25 (1.50) | 3.84 (1.21) | 4.31 (1.02) | 0.46 (1.53) |
| Q3. Can NoV be transmitted through water? | 3.95 (1.13) | 4.04 (1.20) | 0.09 (1.50) | 3.48 (1.35) | 4.02 (1.24) | 0.53 (1.7) |
| Q4. Is summer and autumn have more NoV related outbreaks in Shenzhen? | 2.10 (1.06) | 2.12 (1.25) | 0.02 (1.51) | 3.03 (1.37) | 2.76 (1.47) | -0.27 (2.3) |
| Q5. Is the average incubation period of NoV infectious diarrhea 1-2 days? | 4.05 (0.96) | 4.30 (0.91) | 0.25 (1.20) | 3.76 (1.18) | 4.17 (1.09) | 0.41 (1.55) |
| Q6. Can medicinal alcohol inactivate NoV? | 2.60 (1.29) | 2.96 (1.51) | 0.36 (1.77) | 2.57 (1.35) | 3.69 (1.40) | 1.12 (1.97) |
| Q7. Is vaccination effective in preventing NoV-infected diarrhea? | 2.25 (1.21) | 2.60 (1.49) | 0.34 (1.72) | 2.72 (1.35) | 3.30 (1.50) | 0.59 (2.14) |
| Q8. Will virus excretion in the short term after the symptoms of diarrhea caused by NoV have resolved? | 3.98 (0.98) | 4.16 (1.00) | 0.19 (1.25) | 3.82 (1.06) | 4.20 (1.01) | 0.38 (1.39) |
| Score of knowledge | 24.59 (2.97) | 26.02 (3.97) | 1.44 (3.99) | 26.68 (4.21) | 29.05 (5.14) | 2.37 (6.57) |
| Q9. Are you interested in learning about NoV infectious diarrhea? | 4.18 (0.81) | 4.37 (0.75) | 0.19 (0.95) | 4.37 (0.69) | 4.57 (0.64) | 0.2 (0.89) |
| Q10. The role of faculty in epidemic prevention and control (non-treatment) is second only to that of school doctors | 1.95 (1.02) | 1.87 (1.03) | -0.08 (1.29) | 3.14 (1.42) | 2.92 (1.62) | -0.21 (2.43) |
| Q11. It is necessary for teachers to disclose the epidemic information to parents at any time when the epidemic occurs | 4.03 (1.01) | 4.15 (1.00) | 0.12 (1.25) | 3.79 (1.15) | 4.07 (1.11) | 0.28 (1.53) |
| Q12. The role of morning check in the prevention of infectious diseases is general | 2.90 (1.38) | 3.09 (1.48) | 0.19 (1.76) | 2.58 (1.43) | 3.47 (1.59) | 0.89 (2.24) |
| Q13. Faculty members (except school doctors) need to identify the epidemic in time | 4.32 (0.78) | 4.48 (0.69) | 0.16 (0.89) | 4.41 (0.75) | 4.63 (0.64) | 0.22 (0.93) |
| Q14. Do you agree that teachers or childminders are the first responders to NoV diarrhea outbreaks? | 3.76 (1.13) | 3.84 (1.18) | 0.08 (1.46) | 3.99 (1.04) | 4.33 (0.93) | 0.34 (1.33) |
| Q15. The school closure guideline for epidemic prevention and control are too strict | 3.11 (1.26) | 3.34 (1.30) | 0.22 (1.55) | 2.73 (1.29) | 3.44 (1.33) | 0.7 (1.96) |
| Q16. The impact of school suspension on students is greater than the disease itself | 3.33 (1.30) | 3.65 (1.31) | 0.32 (1.55) | 2.44 (1.31) | 3.69 (1.36) | 1.25 (2.15) |
| Score of Attitude | 27.59 (3.97) | 28.79 (4.10) | 1.20 (4.02) | 27.45 (3.92) | 31.12 (4.42) | 3.67 (5.61) |
| Q17. When parents have objections to school suspension, will you take the initiative to explain the guideline? | 4.26 (0.75) | 4.45 (0.70) | 0.19 (0.86) | 4.32 (0.73) | 4.55 (0.66) | 0.23 (0.89) |
| Q18. Will you take the initiative to point out the improperly dispose of vomit and excrement? | 4.31 (0.73) | 4.48 (0.66) | 0.17 (0.81) | 4.43 (0.65) | 4.65 (0.52) | 0.22 (0.75) |
| Q19. When parents state their children have vomiting or diarrhea at home, will you inform them to take their child home for observation? | 4.30 (0.75) | 4.50 (0.70) | 0.19 (0.88) | 4.42 (0.76) | 4.68 (0.55) | 0.26 (0.86) |
| Q20. When there is a cluster epidemic in the class, do you need to take the initiative to discuss with the school doctor whether to suspend classes? | 4.36 (0.73) | 4.49 (0.68) | 0.13 (0.83) | 4.46 (0.70) | 4.64 (0.62) | 0.17 (0.83) |
| Q21. Is the correct way to deal with the vomit of students in the class to wipe it before disinfecting it? | 2.73 (1.46) | 3.00 (1.62) | 0.27 (1.93) | 2.43 (1.49) | 3.78 (1.54) | 1.35 (2.32) |
| Q22. When a diarrhea outbreak occurs, will you communicate with parents about your child's condition? | 4.38 (0.68) | 4.54 (0.61) | 0.16 (0.77) | 4.45 (0.66) | 4.67 (0.55) | 0.22 (0.79) |
| Q23. Will you judge whether the children coming to school meet the quarantine time according to the absence record? | 4.10 (0.90) | 4.29 (0.88) | 0.19 (1.14) | 3.98 (1.10) | 4.40 (0.89) | 0.42 (1.3) |
| Q24. When a student has unexplained vomiting, do you need to notify parents to take them back? | 4.31 (0.77) | 4.50 (0.68) | 0.19 (0.87) | 4.44 (0.67) | 4.64 (0.60) | 0.21 (0.83) |
| Q25. Do you pay more attention to students' gastrointestinal symptoms than respiratory symptoms in the morning check? | 4.06 (0.84) | 4.18 (0.83) | 0.11 (1.09) | 3.39 (1.27) | 2.66 (1.41) | -0.73 (2.33) |
| Q26. Will you let the student stay in the class after a sudden vomiting? | 3.01 (1.30) | 3.27 (1.38) | 0.26 (1.57) | 2.54 (1.33) | 3.65 (1.36) | 1.12 (2.12) |
| Q27. Can students infected with NoV be allowed to return to school 48 hours after symptoms disappear? | 2.57 (1.29) | 2.58 (1.44) | 0.00 (1.60) | 2.7 (1.43) | 2.84 (1.58) | 0.14 (1.98) |
| Q28. Will you report the situation to the school doctor when you know that more than one classes have children with diarrhea and vomiting during the same period? | 4.46 (0.69) | 4.59 (0.61) | 0.13 (0.77) | 4.51 (0.66) | 4.72 (0.51) | 0.22 (0.76) |
| Score of practice | 46.85 (6.03) | 48.86 (5.86) | 2.01 (5.48) | 46.05 (4.81) | 49.88 (4.78) | 3.83 (5.89) |
| Overall score of KAP | 99.03 (10.07) | 103.68 (10.99) | 4.65 (7.76) | 100.18 (8.79) | 110.05 (10.75) | 9.87 (10.94) |

Note：All values are shown in Mean (SD); D-value means score difference.

| **Table S2** Univariate analysis of factors of demographic by difference in scores before and after intervention | | | | | | | | | | | | |
| --- | --- | --- | --- | --- | --- | --- | --- | --- | --- | --- | --- | --- |
| variable | kindergarten（N=1095） | | | | | | school（N=1028） | | | | | |
|  | K Median(IQR) | *P* | A Median(IQR) | *P* | P Median(IQR) | *P* | K Median(IQR) | *P* | A Median(IQR) | *P* | P Median(IQR) | *P* |
| Nature of school | |  |  |  |  |  |  |  |  |  |  |  |
| Public | 1.00 (-3.00, 7.00) | 0.335 | 4.00 ( 0, 8.00) | 0.199 | 4.00 ( 0, 8.00) | 0.092 | 0 (-1.00, 3.00) | 0.696 | 0 (-1.00, 3.00) | 0.064 | 0 (-2.00, 5.00) | 0.337 |
| Private | 2.00 (-3.00, 8.00) |  | 4.00 ( 0, 7.00) |  | 3.00 ( 0, 7.50) |  | 0 ( 0, 3.00) |  | 1.00 (-1.00, 4.00) |  | 1.00(-2.00, 6.00) |  |
| Sex |  |  |  |  |  |  |  |  |  |  |  |  |
| Male | 2.50 (-1.50, 8.00) | 0.608 | 2.00 (-0.25, 6.50) | 0.500 | 2.50 (-1.00, 8.25) | 0.639 | 0 (-1.00, 2.00) | 0.018* | 0 (-1.00, 3.00) | 0.546 | 0 (-2.00, 5.00) | 0.205 |
| Female | 1.00 (-3.00, 7.00) |  | 4.00 ( 0, 8.00) |  | 4.00 ( 0, 8.00) |  | 0 ( 0, 3.00) |  | 1.00 (-1.00, 4.00) |  | 1.00 (-1.00, 6.00) |  |
| Age |  |  |  |  |  |  |  |  |  |  |  |  |
| ≤25 | 1.00 (-3.00, 7.00) | 0.320 | 4.00 ( 0, 6.25) | 0.576 | 4.00 ( 0, 8.00) | 0.786 | 1.00 (-0.50, 3.00) | 0.413 | 0 (-1.00, 3.50) | 0.314 | 1.00 (-2.00, 6.00) | 0.941 |
| 26-35 | 2.00 (-3.00, 8.00) |  | 4.00 ( 0, 8.00) |  | 4.00 ( 0, 8.00) |  | 0 (-1.00, 3.00) |  | 1.00 (-1.00, 4.00) |  | 1.00 (-2.00, 5.00) |  |
| >35 | 1.00 (-3.00, 7.00) |  | 4.00 (-1.00, 8.00) |  | 4.00 ( 0, 8.00) |  | 0 (-1.00, 4.00) |  | 1.00 (-1.00, 4.00) |  | 1.00 (-1.00, 5.00) |  |
| Position |  |  |  |  |  |  |  |  |  |  |  |  |
| Teacher | 1.00 (-3.00, 7.00) | 0.666 | 4.00 ( 0, 7.00) | 0.843 | 4.00 ( 0, 8.00) | 0.243 | 0 (-1.00, 3.00) | 0.103 | 1.00 (-1.00, 4.00) | 0.018* | 1.00 (-1.75, 6.00) | 0.632 |
| Administrator | 1.00 (-3.00, 8.00) |  | 4.00 ( 0, 8.00) |  | 3.00 (-1.00, 6.00) |  | 1.50 ( 0, 5.75) |  | 0 (-4,.00 2.75) |  | 0.50 (-3.00, 5.50) |  |
| Childminder | 2.00 (-3.00, 7.00) |  | 4.00 ( 0, 8.00) |  | 4.00 ( 0, 8.00) |  | - |  | - |  | - |  |
| Length of service | |  |  |  |  |  |  |  |  |  |  |  |
| ≤5 years | 2.00 (-3.00, 8.00) | 0.843 | 3.00 ( 0, 7.00) | 0.021* | 4.00 ( 0, 8.00) | 0.133 | 0 ( 0, 3.00) | 0.782 | 1.00 (-1.00, 3.75) | 0.839 | 1.00 (-2.00, 5.75) | 0.869 |
| 5-10 years | 1.00 (-3.00, 7.00) |  | 4.00 ( 0, 8.00) |  | 4.00 ( 1.00, 8.00) |  | 0 (-1.00, 3.00) |  | 1.00 (-1.00, 4.00) |  | 1.00 (-2.00, 6.00) |  |
| >10 years | 1.00 (-3.00, 8.00) |  | 4.00 ( 0, 8.00) |  | 3.00 (-1.00, 8.00) |  | 0 (-1.00, 3.00) |  | 1.00 (-1.00, 4.00) |  | 1.00 (-1.00, 5.00) |  |
| Learning stage | |  |  |  |  |  |  |  |  |  |  |  |
| Secondary schools | - |  | - |  | - |  | 1.00 ( 0, 4.00) | <0.001* | 1.00 (-1.00, 4.00) | 0.090 | 2.00 (-1.00, 6.00) | 0.013* |
| Primary schools | - |  | - |  | - |  | 0 (-1.00, 2.00) |  | 0 (-1.00, 3.00) |  | 0 (-2.00, 4.00) |  |

Note: Mann-Whitney U test for two groups and the Kruskal-Wallis test for three groups, K, A, and P denote knowledge, attitude, practice, respectively; *means *p*<0.05.
